# Physicians’ perspectives and future vision on disability assessments by phone during the COVID-19 pandemic: a cross-sectional survey

**DOI:** 10.1186/s12913-022-08068-1

**Published:** 2022-05-23

**Authors:** Nadia Baart, Jan Lucas Hoving, Birgit Helena Petra Maria Donker-Cools

**Affiliations:** 1 Social Security Institute, Social Medical Affairs division, PO Box 58285, 1040 HG Amsterdam, the Netherlands; 2 Coronel Institute of Occupational Health, Department of Public and Occupational Health, Amsterdam UMC and Research Center for Insurance Medicine, PO Box 22700, 1100 DE Amsterdam, the Netherlands

**Keywords:** Telephone [MeSH], Phone consultations, Telemedicine [MeSH], COVID-19 [MeSH], Communication [MeSH], Disability assessment

## Abstract

**Background:**

Physicians, who perform disability assessments for the Dutch Social Security Institute, were urged to conduct phone consultations from their homes to prevent the spread of COVID-19. The purpose of the study was to evaluate the perspectives of physicians regarding phone consultations during the COVID-19 pandemic. Additionally, to explore physicians’ views on a more widespread future use of phone consultations in the context of work disability assessments.

**Methods:**

An electronic survey conducted from June to August 2020 included 41 statements categorized into themes previously identified in both the literature on physicians’ phone consultations and emerging from daily practice. All 1081 physicians working at the Dutch Social Security Institute were invited by e-mail to participate in the survey. Participants indicated on a 5-point Likert scale whether they strongly disagreed, disagreed, neither agreed nor disagreed, agreed or strongly agreed with the statements. The collected data were analysed using descriptive statistics.

**Results:**

In general, physicians had become accustomed to perform phone consultations. Negative experiences included difficulties in getting an impression of patients and assessing patients’ functional limitations. About half of physicians found that phone consultations took more effort, 61% asked more questions due to no direct patient observations. According to 67%, it is mostly necessary to perform an in-person consultation to adequately assess functional limitations of a patient with persistent medically unexplained physical symptoms. A great majority did not prefer telephone consultations to in-person consultations. However, more than half of physicians perceive a greater preference for phone consultations in the future than previously. 56% thought that replacement of in-person consultations with phone consultations in the future might lead to more complaints.

**Conclusions:**

Perspectives and future views varied among physicians performing disability assessments by phone. A majority of physicians experienced difficulties with different aspects of the assessment. Despite these difficulties, most physicians support to continue the wider use of phone consultations. To improve remote disability assessments it is required to gain more insights into conditions under which a phone assessment can be as diligent as an in-person assessment.

## Background

The coronavirus disease (COVID-19) pandemic caused a tremendous health crisis worldwide. Governments across the world introduced strict public health measures to prevent the spread of COVID-19. Following these measures, employees adhered to strict social distancing rules and had to adapt to working from home as much as possible.

In line with national and international healthcare policies to minimize in-person contact with patients whenever possible, [1, 2] all in-person disability assessments of potentially vulnerable disability benefit claimants in the Netherlands were suspended to limit the risk of contagion. Physicians, who perform disability assessments for the Dutch Social Security Institute (SSI), were thus urged to conduct phone consultations from their homes. These measures ensured that sick-listed and chronically ill people had continued and legitimate access to compensation for income loss.

The rapid shift to full-scale phone consultation meant a significant change in the daily routine of physicians working at the Dutch SSI. They were not used to phone consultations on such a large scale and expressed their concerns, [3] particularly about the impact it might have on the quality of their work. According to Dutch professional standards, assessments should be based on adequate methods [4, 5]. These methods comprise, for example, taking a social and medical history of the patient, and performing a physical examination, with the patient as the most important direct source of information. Through these methods physicians are able to gather information about the actual complaints of their patients and how the medical problems affect daily life functioning and work participation of these patients. Physicians questioned if they would be able to conclude an assessment by phone, specifically when patients have persistent medically unexplained symptoms or might disagree with the assessment outcome.

Due to the ongoing COVID-19 pandemic, phone consultations were expanded to ensure continuance of social security services. The rapid implementation of new working methods could present challenges such as missing of non-verbal communication, [6] but it might also provide opportunities regarding accessibility, service efficiency and workload reduction [7, 8].

In order to address challenges and to utilize opportunities, insight in the experiences of physicians who conduct phone consultations is needed. Alternatives to in-person consultation were explored worldwide in many medical fields, [2, 9, 10] but not yet in the field of work disability assessments. It is not known how physicians in this field experience phone consultations. The COVID-19 pandemic and the large number of disability assessments by phone provide an opportunity to obtain insight into how physicians react to phone consultations. This, by investigating the experiences of physicians using this alternative to in-person consultation and to evaluate their perspectives on performing phone consultations in the future. With these findings of this study we hope to inform the potential further use and implementation of phone consultations in the field of work disability assessments.

The aim of this study is:

1. To evaluate the perspectives of physicians regarding phone consultations as an alternative to in-person consultations during the COVID-19 pandemic.
2. To explore physicians’ views on a more widespread future use of phone consultations in the context of work disability assessments.

## Methods

### Design

A cross-sectional survey study was performed in order to evaluate the perspectives of physicians regarding phone consultations and to explore their views on a more widespread future use. The study was reported in accordance to the Checklist for Reporting Results of Internet E-surveys (CHERRIES) [11, 12].

### Participants

The target population comprised physicians employed by the Dutch SSI. The Dutch SSI is an autonomous authority and is commissioned by the Ministry of Social Affairs and Employment to implement employee insurances among other things. One of the core tasks of the Dutch SSI is evaluating illness and labour incapacity. Its 1106 physicians perform disability assessments of patients who apply for a disability benefit after 2 years of sick leave. These assessments comprise an interview with the patient and for example a physical examination or a request for more medical information from third parties. In addition, the physician assesses the patient’s work limitations and abilities. Based on the physician’s assessment the patient may be urged to return to work or receives a disability benefit.

Neither patients, nor any patient data were used in this study.

### Procedure

All 1081 physicians employed by the Dutch SSI were invited to participate; only the 25 physicians who piloted a first version of the survey were excluded. The physicians were informed about the study aim and procedure on June 19, 2020. They were then asked to complete an anonymous online survey that was distributed by email on June 23, 2020; a reminder was sent 6 weeks later. Both emails contained a link to the online survey tool Metrics that Matter. Physicians who decided to participate provided informed consent in the first survey question. Answers could be changed until survey submission; results were only saved after submission. The survey tool avoided duplicate entries from the same email address. The survey was closed on August 18, 2020.

### Survey

The research team developed a first version of the survey, based on experiences with phone consultations in healthcare, identified by scoping the scientific literature, and adapted to the context of work disability assessments [6–8, 13–15]. This first version of the survey was piloted by 25 physicians at the Dutch SSI. These physicians provided feedback regarding the content validity and readability of the survey. The research team adapted the survey on the basis of the feedback and suggestions for improvement. The final survey contained 41 statements categorized into seven topics: physician-related aspects, legal aspects, practical aspects, medical aspects, patient-related aspects, communication aspects and phone consultations in the future. Physicians indicated on a 5-point Likert scale whether they strongly disagree, disagree, neither agree nor disagree, agree or strongly agree with the statements. A ‘not applicable’ answer choice was added. In addition, physicians rated the phone consultations on the whole on a scale from 1 (very bad) to 10 (very good). Finally, physicians’ demographic data on age, gender and working experience were collected.

### Data analysis

Descriptive statistical analysis of physicians’ agreement or disagreement with the statements was performed, using SPSS statistics, version 26. The response options were dichotomised, indicating either agreement (strongly agree, agree) or no agreement (other three categories) with each statement. The number of physicians and percentages were reported. In addition, for the last question “On the whole, I would rate the phone consultations as follows” the mean and the median rates, including the interquartile range (IQR) were presented.

## Results

A total of 345 physicians responded to the survey. Four of them did not provide informed consent. Three hundred forty-one physicians completed the survey, resulting in a response rate of 32%. Fifty-three percent were female, the mean age was 48 years (SD 14) and 60% were senior physicians. A range of working experience was covered: 50% worked less than 10 years for the Dutch SSI, 37% more than 20 years.

More than half (59%) of the physicians rated the phone consultations with a 7 or higher on a scale of 1 to 10. The mean rate was 6.4 and the median rate was 7 (IQR 6–8).

### Physician-related aspects

The majority of the physicians (66%) found that phone consultations came easily to them and 74% experienced getting used to them (Table 1). On the other hand, only 19% preferred holding phone consultations to holding in-person consultations. Half of physicians (50%) felt they had more space to organize their own day when holding phone consultations, and 24% experienced less work pressure.

**Table 1:** Physicians’ experiences

	Total (N)	(Strongly) agree (%)
1: On the whole, I find that phone consultations come easily to me.	336	66%
2: I have become used to holding phone consultations.	336	74%
3: On the whole, I prefer holding phone consultations to holding in-person consultations.	328	19%
4: I experience less work pressure when holding phone consultations than when holding in-person consultations.	328	24%
5: I have more space to organize my own day when holding phone consultations than when holding in-person consultations.	334	50%
6: I am more worried about receiving a complaint/disciplinary complaint if I replace an in-person consultation with a phone consultation.	338	43%
7: I think that phone consultations will lead to more objections and appeal cases against the assessment outcomes.	332	59%
8: I am discussing the assessment with another physician (supervisor or peer) more often than I normally would.	318	14%
9: I am consulting with a colleague more often than I normally would.	326	16%
10: I am unable to conclude an assessment by phone if the patient does not agree with my verdict.	321	29%
11: The possibility that the patient may be recording the conversation makes me feel uncomfortable.	336	34%
12: I have concerns about my patient’s privacy when doing phone consultations.	334	14%
13: I find that a conversation in a phone consultation takes me more time than an in-person consultation.	334	32%
14: I find that reporting when doing a phone assessment takes me more time than reporting after an in-person consultation.	332	25%
15: I find that phone consultations take more effort on my part than in-person consultations.	334	49%
16: I find that patients are easy to reach for the phone consultations.	330	67%
17: I often experience problems with the connection during phone consultations	334	24%
18: I find it difficult to get an impression of someone without the observations of an in-person consultation.	336	59%
19: I ask more questions in order to get an impression during a phone consultation.	335	61%
20: I ask for more medical information from third parties than I normally would.	333	48%
21: When doing a phone consultation, I go along with a patient’s claim more than I normally would.	330	51%
22: I find it difficult to assess functional limitations when I have not seen a patient at an in-person consultation.	320	56%
23: I have no problem with removing a functional limitation given at an earlier assessment without seeing the patient at an in-person consultation.	313	28%
24: With a musculoskeletal disorder, I find that it is mostly necessary to hold an in-person consultation to establish the functional limitations.	339	66%
25: With a psychological disorder, I find that it is mostly necessary to hold an in-person consultation to establish the functional limitations.	338	36%
26: With persistent medically unexplained physical symptoms, I find that it is mostly necessary to hold an in-person consultation to establish the functional limitations.	335	67%
27: With a combination of physical and psychological disorders, I find that it is mostly necessary to hold an in-person consultation to establish the functional limitations.	339	64%
28: I have good contact with my patients during a phone consultation.	333	83%
29: I get the impression that patients are satisfied with a phone consultation as an alternative.	329	71%
30: If there is a language barrier, I find that it is feasible to do a phone consultation with an interpreter who has dialled in.	182	28%
31: I find that it is feasible to do a phone consultation when a supervisor has dialled into the conversation.	266	45%
32: I find it more difficult to ask sensitive questions during a phone consultation.	335	32%
33: I find it more difficult to assess whether a patient has understood me during a phone consultation.	337	55%
34: I am less afraid of aggression during a phone consultation.	319	48%
35: I find it easier to communicate bad news by phone.	323	24%

*N* number, % = percentage of physicians who agree or strongly agree with the statement

### Legal aspects

Forty-three percent of the physicians were more worried about receiving a complaint by replacing in-person consultations with phone consultations. Furthermore, 59% thought it would lead to more objections and appeal cases against the outcome of the assessment. On the other hand, only a minority (14%) discussed the assessment with a supervisor or peer. Twenty-nine percent of the physicians were unable to conclude an assessment by phone if the patient disagreed with their verdict. Few physicians (14%) had concerns about their patients’ privacy.

### Practical aspects

Almost half of the physicians (49%) felt that phone consultations took more effort on their part than in-person consultations. The conversations and the reporting took more time for 32 and 25%, respectively.

Patients were usually easy to reach by phone according to 67% of the physicians; few (24%) had problems with the connection.

### Medical/socio-medical aspects

Concerning the socio-medical assessment, 59% of the physicians had difficulties getting an impression of their patients without the observations made in an in-person consultation and 61% asked more questions to get an impression. About half (48%) asked more frequently for more medical information from third parties. Also, 51% went along with their patient’s claim more than they usually would and 56% found it difficult to assess functional limitations without seeing the patient at an in-person consultation.

### Patient-related aspects

A majority of the physicians found that an in-person consultation is mostly necessary to assess functional limitations: specifically in cases with persistent medically unexplained physical symptoms (67%), musculoskeletal disorders (66%) and a combination of physical and psychological disorders (64%). A minority (36%) of the physicians found in-person consultations mostly necessary for assessing psychological disorders. The vast majority (83%) had good contact with their patients by phone and 71% felt that patients were satisfied with this alternative.

### Communication aspects

Few (28%) physicians found that a phone consultation with an interpreter was feasible if there was a language barrier. Less than half (45%) found that it was feasible when a supervisor dialled into the conversation. Furthermore, 55% found it more difficult to assess whether a patient understood them during a phone consultation. Almost half (48%) of the physicians indicated that they were less afraid of aggression during a phone consultation and 24% found it easier to communicate bad news by phone.

### Phone consultations in future

More than half (55%) of the physicians would like to continue to do more phone consultations than they used to and 52% support the idea that this kind of working should form part of their future working method (Table 2). Furthermore, 59% thought that the Dutch SSI has enough facilities to focus more on phone consultations in the future. A majority (56%) answered they are better at studying the files to establish which patients can be assessed by phone thanks to gained experience. On the other hand, 56% also indicated there might be an increased chance of receiving a complaint if they replace an in-person consultation with a phone consultation when in-person consultations become possible again in future.

**Table 2:** Phone consultations in the future

	Total (N)	(Strongly) agree (%)
36: Even after the coronavirus crisis, I would like to continue to do more phone consultations than we used to.	337	55%
37: I support the idea that this kind of working should form part of our future working method.	339	52%
38: Thanks to the experience I have gained with phone consultations, I am better at studying the files to establish who can be assessed by phone as an alternative to an in-person consultation, including after the coronavirus crisis.	332	56%
39: When in-person consultations become possible again in future, I think there will be a higher chance of receiving a complaint/disciplinary complaint if I replace an in-person consultation with a phone consultation.	333	56%
40: I think that the Dutch SSI has enough facilities to focus more on phone consultations in the future.	325	59%
41: I would prefer to go back completely to my previous way of working.	336	39%

*N* number, % = percentage of physicians who agree or strongly agree to the statement

## Discussion

The aim of this study was to explore physicians’ experiences with disability assessments by phone during the COVID-19 pandemic and their perspectives regarding phone consultations in the future. Overall, 74% of the physicians have become used to holding phone consultations, 66% found them easy to perform and 83% experienced good contact with their patients. On the other hand, more than half (56%) experienced difficulties in assessing functional limitations by phone, and 59% were worried about legal consequences. Forty-nine percent felt that phone consultations took more effort on their behalf. A great majority (81%) does not prefer phone consultations to in-person consultations, but 55% would like to perform more phone consultations in the future than they were previously used to.

About half of the physicians (51%) reported going along more often with their patients’ disability claim when holding phone consultations. A possible explanation is that the physicians tend to base the conclusion of disability assessments more on self-reported limitations of the patients, as a thorough clinical evaluation by phone is hindered. In a study comparing self-reported limitations with those derived from clinical evaluation, self-reported limitations were found to be considerably higher [16]. In the current study, in-person consultations were considered particularly important for patients with physical disorders and medically unexplained physical symptoms. At the same time, the absence of direct contact challenged physicians in forming a complete impression and may explain the extra time spent questioning patients and the more frequent requests for more medical information from third parties. In a study by Chaudhry et al. general practitioners reported similar negative experiences with phone consultations linked to the absence of non-verbal cues, difficulty in picking up cues, the absence of a physical examination and difficulty in more complex consultations [17]. Yildiz et al. reported that for follow-up appointments in an oncology department a phone consultation was sufficient in 32% of cases [18]. In these cases, no additional examination or intervention was necessary [18]. In conclusion, it may be necessary to hold in-person consultations with new patients, complex disorders and when a physical examination is needed.

The challenges of phone consultations experienced by the physicians in the current study may to some degree be related to its sudden full-scale implementation. This study was performed during the onset of the COVID-19 pandemic when physicians had to adjust to a new way of assessment and to working from home. Over time, physicians may grow accustomed to holding phone consultations. Training ‘on-the-job’ or practical experience may impact its success over time. For example, confidence in phone consultations in general practitioner trainees was linearly related to received training [17]. A study among psychiatry trainees suggested that more experience with the use of telepsychiatry was associated with fewer and less stronger concerns about using it [19]. Training programmes could possibly also improve physicians’ experiences with phone consultations at the Dutch SSI.

The current increasing interest in phone consultation as an alternative to in-person consultation is related to the aim to improve patient access to healthcare while also improving efficiency in managing the workload of healthcare professionals, [6–8, 14, 15] particularly in times of a growing lack of personnel [13].

The lack of physicians and high work demands are a well-recognized problem in the field of disability assessments in the Netherlands. This study showed that a minority of physicians experienced less work pressure when holding phone consultations. Although not investigated in this study, some physicians may experience more stress. This could be related to work-family role blurring as physicians were required to work from their homes during the COVID-19 pandemic [20, 21]. In addition, it was shown that taking care of children due to school closure was as a primary source of conflict that hinders teleworking [22].

This study did not investigate satisfaction as an outcome, but 59% of the physicians rated phone consultations with a seven out of ten, which could be an indication of overall satisfaction. In previous studies, physicians in other medical fields were satisfied with alternatives of in-person consultations during COVID-19 [23, 24]. A systematic review found no differences in surgeon satisfaction when comparing telemedicine including phone consultations, video consultations or internet-based care, with in-person care for orthopaedic assessments [23]. Another study reported that overall, sports medicine physicians felt satisfied with video consultations [24].

Video consultations were rapidly adopted in other medical fields and studies demonstrated that video consultations could be a useful option in the field of neurology, neurosurgery and orthopaedic surgery, where detailed physical examinations are frequently needed [25–28]. Franco et al. reported that the use of telemedicine in spinal practice was feasible [26]. A virtual exam successfully replaced the traditional physical exam. Virtual tests comprised visual inspection and functional tests but strength could also be objectively tested using household objects of known weight [26, 28]. However, the dependence on the patient to elicit findings during assessment could be considered a challenge of telemedicine, [26] especially in the field of disability evaluation.

This study explored the experiences and future perspectives of physicians regarding disability assessments by phone. A limitation of this study is that patient perspectives were not evaluated. It is important to investigate how patients perceive remote assessments, to assure sustainability of this approach. A study among patients with cancer suggested that a majority of patients with cancer prefer in-person consultations, although 38% are still willing to have a phone or video consultation again in the future [29]. Another survey study reported 42% of patients of sports medicine physicians preferred a video consultation in the absence of COVID-19 restraints [24]. Findings among psychiatric patients suggested that 64% considered using remote treatment sessions (phone or video) in the future [30]. Thus, a considerable number of patients may be willing to adapt to current developments.

This study demonstrated the difficulties physicians have in assessing functional limitations by phone, for example in a patient with persistent medically unexplained physical symptoms. Therefore, it is important to understand the conditions under which a phone assessment can be as diligent as an in-person assessment.

A good triage system could improve the suitability and efficiency of phone consultations. In this study, only a minority of the physicians found that it is mostly necessary to hold an in-person consultation in the case of a psychosocial disorder. By contrast, it was found that an in-person consultation may well be more suitable for patients with persistent medically unexplained physical symptoms. We recommend investigating which patients are suitable for phone consultations. In addition, a more standardized approach could improve the reliability of assessments [31].

A bottleneck in conducting phone consultations is the lack of visual information; video consultation could provide a solution in some cases. Video consultations do not allow for a complete examination, but methods useful for assessing functional limitations could be devised for the field of work disability assessments comparable to the neurologic and orthopaedic surgery fields.

Finally, it could be useful to explore the patients’ perspective on phone and video consultation regarding disability assessments in future studies, as this is not yet known. This could identify which patients would benefit from phone and video consultation, as there is a high diversity of both physical and mental disorders in the group of disability benefit claimants.

## Conclusions

This study demonstrated that physicians’ experiences and future perspectives regarding disability assessments by phone varied. A majority finds it difficult to get an impression of a patient and to assess functional limitations. However, also a majority supports to continue the wider use of phone consultations and the idea that phone consultations should form part of their future working method.

This study provides a starting point to further evaluate and adjust available methods in order to further improve remote disability assessments. This includes exploring the stakeholder perspectives of patients and their experiences with phone consultations during disability assessments. In addition, it will be interesting to see how the experience gained during the COVID-19 pandemic will change organizational policies and work processes, and affect the need for remote disability assessments in the long term.

## Data Availability

The datasets generated during and/or analysed during the current study are not publicly available due to privacy restrictions as the data contain information that could compromise the privacy of research participants, but are available from the corresponding author on reasonable request.

## Abbreviations

CHERRIES: Checklist for Reporting Results of Internet E-surveys
COVID-19: Coronavirus disease
SSI: Social Security Institute

## References

[1] Ohannessian R, Duong TA, Odone A (2020). Global telemedicine implementation and integration within health systems to fight the COVID-19 pandemic: a call to action. JMIR Public Health Surveill.

[2] Webster P (2020). Virtual health care in the era of COVID-19. Lancet..

[3] Weel I (2020). Via de telefoon bepalen of iemand wel of niet kan werken, niet mogelijk zeggen UWV-artsen..

[4] Knepper JF, Faas JWA (2000). Inleiding Verzekeringsgeneeskundige beoordeling. Nederlandse Vereniging voor Verzekeringsgeneeskunde.

[5] Landelijk instituut sociale verzekeringen (2000). Onderzoeksmethoden standaard. Nederlandse Vereniging voor Verzekeringsgeneeskunde.

[6] Atherton H, Brant H, Ziebland S, Bikker A, Campbell J, Gibson A (2018). Alternatives to the face-to-face consultation in general practice: focused ethnographic case study. Br J Gen Pract.

[7] Downes MJ, Mervin MC, Byrnes JM, Scuffham PA (2017). Telephone consultations for general practice: a systematic review. Syst Rev.

[8] Bunn F, Byrne G, Kendall S. Telephone consultation and triage: effects on health care use and patient satisfaction. Cochrane Database Syst Rev. 2004:CD004180. 10.1002/14651858.CD004180.pub2. 10.1002/14651858.CD004180.pub2 15495083

[9] Bokolo AJ (2020). Use of telemedicine and virtual Care for Remote Treatment in response to COVID-19 pandemic. J Med Syst.

[10] Wosik J, Fudim M, Cameron B, Gellad ZF, Cho A, Phinney D (2020). Telehealth transformation: COVID-19 and the rise of virtual care. J Am Med Inform Assoc.

[11] Turk T, Elhady MT, Rashed S, Abdelkhalek M, Nasef SA, Khalaff AM (2018). Quality of reporting web-based and non-web-based survey studies: what authors, reviewers and consumers should consider. PLoS One.

[12] Eysenbach G (2004). Improving the quality of web surveys: the checklist for reporting results of internet E-surveys (CHERRIES). J Med Internet Res.

[13] Brant H, Atherton H, Ziebland S, McKinstry B, Campbell JL, Salisbury C (2016). Using alternatives to face-to-face consultations: a survey of prevalence and attitudes in general practice. Br J Gen Pract.

[14] Campbell JL, Fletcher E, Britten N, Green C, Holt TA, Lattimer V (2014). Telephone triage for management of same-day consultation requests in general practice (the ESTEEM trial): a cluster-randomised controlled trial and cost-consequence analysis. Lancet..

[15] Hammersley V, Donaghy E, Parker R, McNeilly H, Atherton H, Bikker A (2019). Comparing the content and quality of video, telephone, and face-to-face consultations: a non-randomised, quasi-experimental, exploratory study in UK primary care. Br J Gen Pract.

[16] Brouwer S, Dijkstra PU, Stewart RE, Göeken LNH, Groothoff JW, Geertzen JHB (2005). Comparing self-report, clinical examination and functional testing in the assessment of work-related limitations in patients with chronic low back pain. Disabil Rehabil.

[17] Chaudhry U, Ibison J, Harris T, Rafi I, Johnston M, Fawns T. Experiences of GP trainees in undertaking telephone consultations: a mixed-methods study. BJGP Open. 2020;4. 10.3399/bjgpopen20X101008. 10.3399/bjgpopen20X101008 PMC7330189 32019774

[18] Yildiz F, Oksuzoglu B (2020). Teleoncology or telemedicine for oncology patients during the COVID-19 pandemic: the new normal for breast cancer survivors?. Future Oncol.

[19] Orchard K, Cruz C, Shoemaker EZ, Hilty DM. A survey comparing adult and child psychiatry trainees, faculty, and program Directors' perspectives about telepsychiatry: implications for clinical care and training. J Technol Behav Sci. 2021:1–10. 10.1007/s41347-020-00187-y. 10.1007/s41347-020-00187-y PMC7820828 33501373

[20] Sinclair RR, Allen T, Barber L, Bergman M, Britt T, Butler A (2020). Occupational health science in the time of COVID-19: now more than ever. Occup Health Sci.

[21] Vaziri H, Casper WJ, Wayne JH, Matthews RA (2020). Changes to the work-family interface during the COVID-19 pandemic: examining predictors and implications using latent transition analysis. J Appl Psychol.

[22] Zhang S, Moeckel R, Moreno AT, Shuai B, Gao J (2020). A work-life conflict perspective on telework. Transp Res A Policy Pract.

[23] Chaudhry H, Nadeem S, Mundi R (2021). How satisfied are patients and surgeons with telemedicine in Orthopaedic care during the COVID-19 pandemic? A systematic review and Meta-analysis. Clin Orthop Relat Res.

[24] Kirby DJ, Fried JW, Buchalter DB, Moses MJ, Hurly ET, Cardone DA, et al. Patient and physician satisfaction with Telehealth during the COVID-19 pandemic: sports medicine perspective. Telemed J E Health. 2021. 10.1089/tmj.2020.0387. 10.1089/tmj.2020.0387 33512302

[25] Al Hussona M, Maher M, Chan D, Micieli JA, Jain JD, Khosravani H (2020). The virtual neurologic exam: instructional videos and guidance for the COVID-19 era. Can J Neurol Sci.

[26] Franco D, Montenegro T, Gonzalez GA, Hines K, Mahtabfar A, Helgeson MD, et al. Telemedicine for the spine surgeon in the age of COVID-19: multicenter experiences of feasibility and implementation strategies. Global. Spine J. 2020:2192568220932168. 10.1177/2192568220932168. 10.1177/2192568220932168 PMC8119918 32677513

[27] Loeb AE, Rao SS, Ficke JR, Morris CD, Riley LH, Levin AS (2020). Departmental experience and lessons learned with accelerated introduction of telemedicine during the COVID-19 crisis. J Am Acad Orthop Surg.

[28] Tanaka MJ, Oh LS, Martin SD, Berkson EM (2020). Telemedicine in the era of COVID-19: the virtual Orthopaedic examination. J Bone Joint Surg Am.

[29] van de Poll-Franse LV, de Rooij BH, Horevoorts NJE, May AM, Vink GR, Koopman M, et al. Perceived care and well-being of patients with Cancer and matched norm participants in the COVID-19 crisis: results of a survey of participants in the Dutch PROFILES registry. JAMA Oncol. 2020. 10.1001/jamaoncol.2020.6093. 10.1001/jamaoncol.2020.6093 PMC7689559 33237294

[30] Guinart D, Marcy P, Hauser M, Dwyer M, Kane JM (2020). Patient attitudes toward Telepsychiatry during the COVID-19 pandemic: a nationwide, multisite survey. JMIR Mental Health.

[31] Barth J, de Boer WEL, Busse JW, Hoving JL, Kedzia S, Coubain R (2017). Inter-rater agreement in evaluation of disability: systematic review of reproducibility studies. BMJ..

[32] World Medical Association (2013). World medical association declaration of Helsinki: ethical principles for medical research involving human subjects. JAMA..

